# Facilitating stress prevention in micro and small-sized enterprises: protocol for a mixed method study to evaluate the effectiveness and implementation process of targeted web-based interventions

**DOI:** 10.1186/s12889-022-12921-7

**Published:** 2022-03-26

**Authors:** Miriam Engels, Leif Boß, Judith Engels, Rebekka Kuhlmann, Johanna Kuske, Sarah Lepper, Lutz Lesener, Valeria Pavlista, Mathias Diebig, Thorsten Lunau, Sascha A. Ruhle, Florian B. Zapkau, Peter Angerer, Jörg Hoewner, Dirk Lehr, Christian Schwens, Stefan Süß, Ines C. Wulf, Nico Dragano

**Affiliations:** 1 Institute of Medical Sociology, Centre for Health and Society, Medical Faculty, Heinrich-Heine-University Dusseldorf, Moorenstr. 5, 40225 Dusseldorf, Germany; 2 Department of Work and Organisational Psychology, Faculty of Psychology, Open University of the Netherlands, Valkenburgerweg 177, 6419 AT Heerlen, The Netherlands; 3 Department of Health Psychology and Applied Biological Psychology, Institute of Psychology, Leuphana University Luneburg, Universitätsallee 1, 21335 Luneburg, Germany; 4 Chair of Business Administration, in particular Work, Human Resource Management and Organization Studies, Faculty of Business Administration and Economics, Heinrich-Heine-University Dusseldorf, Universitätsstr. 1, 40225 Dusseldorf, Germany; 5 Endowed Chair for Interdisciplinary Management Science, Faculty of Management, Economics and Social Sciences, University of Cologne, Albertus-Magnus-Platz, 50923 Cologne, Germany; 6 K12 Agentur für Kommunikation und Innovation GmbH, Schirmerstr. 76, 40211 Dusseldorf, Germany; 7 Institute of Occupational, Social and Environmental Medicine, Centre for Health and Society, Medical Faculty, Heinrich-Heine-University Dusseldorf, Moorenstr. 5, 40225 Dusseldorf, Germany; 8 Department of Human Resource Studies, Tilburg University, Prof. Cobbenhagenlaan 225, 5037 DB Tilburg, The Netherlands; 9 Institute for International Business, Department of Global Business and Trade, Vienna University of Economics and Business, Welthandelsplatz 1, 1020 Vienna, Austria

**Keywords:** Web-based intervention, MSE, Stress prevention, Occupational health, Implementation, Process evaluation

## Abstract

**Background:**

Workplace-related stress is a major risk factor for mental and physical health problems and related sickness absence and productivity loss. Despite evidence regarding the effectiveness of different workplace-based interventions, the implementation of stress prevention interventions is rare, especially in micro and small-sized enterprises (MSE) with fewer than 50 employees. The joint research project “PragmatiKK”^+^ aims to identify and address the specific barriers to the implementation of stress prevention interventions in MSE. This study protocol describes a mixed method study design to evaluate the effectiveness of adapted stress prevention interventions and the implementation process via an integrated web-based platform (“System P”) specifically targeted at MSE.

**Methods:**

First, we develop a web-based intervention, which accounts for the specific working conditions in MSE and addresses stress prevention at a structural and behavioral level. Second, we use common methods of implementation research to perform an effect and process evaluation. We analyze the effectiveness of the web-based stress prevention interventions by comparing depressive symptoms at baseline and follow-up (after 6 months and 12 months). Indicators for a successful implementation process include acceptability, adoption, feasibility, reach, dose, and fidelity, which we will measure with quantitative web-based questionnaires and qualitative interviews. We will also analyze the accumulated usage data from the web-based platform.

**Discussion:**

Collecting data on the implementation process and the effectiveness of a web-based intervention will help to identify and overcome common barriers to stress prevention in MSE. This can improve the mental health of employees in MSE, which constitute more than 90% of all enterprises in Germany.

^+^ Full Project Name: „PragmatiKK - Pragmatische Lösungen für die Implementation von Maßnahmen zur Stressprävention in Kleinst- und Kleinbetrieben “(= Pragmatic solutions for the implementation of stress prevention interventions in micro and small-sized enterprises).

**Trial registration:**

German Register of Clinical Studies (DRKS): DRKS00026154, date of registration: 2021-09-16.

**Supplementary Information:**

The online version contains supplementary material available at 10.1186/s12889-022-12921-7.

## Introduction

### Background

Over the past decades, mental health problems have become one of the main causes of sickness absence and early retirement [1–3]. Stress in the workplace (“work-related stress”) is one of the most important psychosocial risk factors, which is also a major risk factor for other health problems (e.g. cardiovascular disease, migraines, muscular pain, etc.) [4, 5]. Despite evidence regarding the effectiveness of workplace-based interventions in preventing work-related stress, the implementation of such interventions in micro and small enterprises (MSE) remains rare [6]. The main goal of the interdisciplinary project “PragmatiKK” is to evaluate the implementation of a comprehensive web-based stress prevention intervention for MSE. This study protocol presents an integrated web-based platform (System P) and describes the proposed mixed-method study for its process and effect evaluation.

### Occupational stress prevention

There are two main approaches of interventions to reduce work-related stress and prevent stress-related illnesses: (1) structural interventions at organizational level and (2) behavioral interventions at individual level. Structural interventions include workplace psychosocial risk-assessment (PRA), workplace rehabilitation measures or all interventions that involve changes in the working conditions of the organization. PRA is an intervention with a special focus on analyzing and reducing psychological hazards and sources of work-related stress (hereinafter referred to as “psychosocial stressor” )[7]. PRA is recommended or even legally required in the European Union [8]. Behavioral interventions help individual employees to develop healthy strategies to cope with stress and include, for example, stress-management trainings (SMT) or other resilience interventions. Experts agree that a combination of both approaches of interventions is the most effective way to achieve the comprehensive and sustainable prevention of work stress [9].

In Germany, less than 20% of all enterprises implement a combination of structural and behavioral interventions for stress prevention [6]. The proportion of enterprises that have implemented PRA is the lowest among MSE [10]. Consequently, employees in MSE often lack access to occupational stress prevention interventions, even though they represent 40% of the total workforce in Germany. Previous research suggests that the reasons for the low implementation rates are complex and underexplored [11, 12]. Generally, there seems to be a low awareness of the benefits of stress prevention in MSE. It has also been shown that MSE tend to have limited knowledge, awareness as well as competence regarding safety and health aspects at work, which are mandatory for implementing these health interventions. An additional barrier is the lack of knowledge about the process of implementing effective stress prevention interventions [13, 14]. This is particularly the case for complex structural interventions such as PRA. Pavlista et al. [14] also found that stigmatization of mental health is a barrier for employers of MSE. Another common inhibitor for MSE are their limited resources for health interventions, i.e. lack of time, staff and money [15–17].

Web-based interventions represent a cost and time-efficient solution for occupational stress prevention [18]. At organizational level, Havermans and colleagues [19] showed that a web-based implementation strategy for PRA could be effective for stress prevention in a large health-care organization. Despite some efforts to develop web-based tools for structural interventions in smaller organizations [e.g. 20, 21], studies of their effectiveness in MSE are still rare. At individual level, web-based SMT allow employees to participate at any time and place with more discretion. Studies show that web-based SMTs are effective in reducing stress and mental health problems (e.g. depressive symptoms) [22]. The greatest effects have been observed for web-based SMT with additional real-time guidance from instructors compared to SMT which are exclusively based on self-learning [18]. Furthermore, guided web-based SMT can still be a cost-effective prevention intervention for employers as they increase productivity [23, 24]. Yet, comprehensive implementation research for web-based occupational stress prevention (including PRA and SMT) in the setting of small businesses is still lacking.

### Implementation of interventions for occupational stress prevention

In addition to the evaluation of effectiveness, implementation research investigates the factors that affect the translation of evidence-based practices into real world settings [25]. To facilitate the implementation of stress prevention interventions in occupational settings, Nielsen and Randall [26] recommend intervention studies that include an extensive process evaluation. Proctor and colleagues [27] summarize eight outcomes for a successful implementation process: acceptability, adoption/reach, appropriateness, feasibility, fidelity/dose, costs, penetration and sustainability.

Acceptability, adoption and appropriateness are important indicators in the early phases of the implementation process. *Acceptability* is defined as the perception among stakeholders that an intervention is desirable and satisfactory [28]. It is a dynamic outcome, as acceptability can change with experience during the implementation process and can be measured with quantitative and qualitative methods. *Adoption* describes the initial decision to implement an intervention and *reach* indicates to what extent the targeted population can be reached (e.g. which MSE sign up for taking part in stress prevention programs) [29]. *Appropriateness* of an intervention describes the perceived fit of an intervention for a specific setting. It differs from acceptability as a stakeholder, e.g. an employer, and can evaluate a stress prevention program as desirable but not appropriate for their organization (e.g. due to lack of resources) [27].

Feasibility, costs and fidelity of the delivery are important indicators after the initial implementation of the intervention. *Feasibility* is related to the concept of appropriateness but measures the actual fit between the intervention and a setting rather than the perceived fit. It is usually assessed retrospectively in order to identify barriers to an effective delivery [30]. The *costs* of the intervention can be direct (e.g. purchase price of training) and indirect (e.g. time and personnel required for training) and should be measured on the side of the adopter and the provider [27]. *Fidelity* describes the extent to which an intervention was implemented as intended, for example in terms of chronological order and *dose* (amount) of the delivered components [29].

Finally, penetration and sustainability are outcomes, which reflect the long-term success of the implementation process. *Penetration* is defined as the extent to which an intervention is integrated into a setting (e.g. do all employees of an organization get access to an intervention) [31]. *Sustainability* is achieved when an organization maintains the intervention after the implementation phase [32].

There are very few implementation studies in the setting of MSE for general prevention programs [33] and to the best of our knowledge, there are currently no previous studies with a comprehensive process evaluation for occupational stress prevention in small companies.

### Study aims

For the purpose of this study, we combine two existing web-based interventions for stress prevention in occupational settings into one integrated web-based platform, called “System P”, specifically targeted at MSE. The aim of the study is to evaluate the effectiveness of the combined web-based interventions for stress prevention and the implementation process via “System P”. In the following, we describe the intervention components of the web-based platform “System P”. Afterwards, we present the protocol for the mixed-method evaluation study.

## Methods

### Part I – Integrated web-based platform “System P”

The web-based platform “System P” includes two adapted interventions for stress prevention: 1) a web-based tool for workplace PRA and 2) a web-based SMT.

*1) Web-based tool for psychosocial risk assessment (“Workplace check”).*

The web-based tool for workplace PRA provides a simplified version of the PRA process according to the German guidelines for structural stress prevention [GDA; 34]. To promote acceptance, we avoid using the term “psychosocial risk assessment” and have replaced it by the less stigmatized term “workplace check”. The workplace check consists of three main steps: (1) Preparation, (2) Analysis and Actions, and (3) Evaluation (see Table 1). During the preparation, employers generate questionnaires for each field of activity in their enterprise to assess possible psychosocial risks. “System P” provides a pool of 55 questions that covers a wide range of psychosocial stressors. The questions cover the main areas of work-related stress (organization, workload, social support, physical environment and boundaries) based on validated PRA questionnaires [20, 35]. Optional items on specific risks related to the COVID-19 pandemic (e.g. fear of infection at work) have been added. The standard pre-selected questionnaire is a short version with nine questions on the most important stressors according to the GDA guidelines. “System P” also includes an option to select all items automatically that are recommended for a full PRA with 43 questions. Employers can select and deselect each item individually and create their own questions for specific risks in their organization. Each item from the questionnaire pool includes a question referring to the occurrence and intensity of a psychosocial stressor, one question asking about possible reasons for the stressor, and a free-text field for suggested solutions to reduce the psychosocial risk. The final PRA questionnaire can be sent to the employees directly via the tool or by copying the URL and sending it for example via email.

**Table 1:** Contents of the web-based tool for workplace psychosocial risk assessment (PRA)

Step^**a**^	Intervention content
1	Preparation of questionnaires
2	Stressor analysis and developing actions
3	Evaluation of actions

Note: ^a^ in the official GDA guidelines, a full PRA cycle includes seven steps which have been summarized but not discarded in “System P” for the purpose of simplification

In the second step, the results of the assessment are summarized for each item individually. A color scheme is used in which areas requiring the action of the employer are indicated in red, moderate areas in yellow, and areas with no need for action in green. This gives employers the opportunity to see which psychosocial stressors are present in their organization and to what extent. The ‘Analysis and Actions’ page also displays possible reasons for each stressor and possible actions to reduce it (employees suggestions provided during the first step and a pre-defined set of actions developed by experts based on scientific findings). Based on this information, employers can create suitable actions and document the implementation process in the tool. “System P” optionally initiates a short survey among employees to rate the potential success of a planned action before its implementation.

After a specific action has been implemented for a reasonable period of time, it is evaluated in the third step. The tool sends an automatic reminder after 3 months of implementation to remind the employer to initiate the evaluation survey. Employees can evaluate the success of the structural interventions and make suggestions for improvement. Their feedback can be used to adjust the action, develop a new one or simply document the success of the action. All steps and results are documented by the tool and can be downloaded during or at the end of the process. The duration of the whole PRA cycle depends strongly on the individual approach of the MSE and can vary between 3 months and 12 months.

*2) Web-based stress management training (“GET.ON Stress”).*

The web-based training “GET.ON Stress” has already been evaluated showing significant effects on stress and stress-related problems like depressive symptoms as compared to controls [36–39]. It was designed to enhance two strategies of stress coping: problem-solving [40] and emotion regulation [41]. The training consists of seven sessions that participants should work on following a weekly schedule [36, 42] (see Table 2). Each session consists of general information, interactive exercises, prototype training participants – so called *personas*– who represent different stressed employee groups, quizzes, audio and video files and downloadable work sheets. In addition, at the end of sessions 2 to 6, users can choose to obtain extra information and perform short exercises about the following common stress-related topics: time management, rumination and worrying, psychological detachment from work, sleep hygiene, sleeping habit rhythm and regularity, nutrition and exercise, organization of breaks during work, and social support [42]. For this trial, we adapted “GET.ON Stress” to employees working at small businesses. Specifically, we adapted the personas within the web-based program who guide participants through the training. Personas are a well-established element of user-centered design in software engineering [43] that has also been used to tailor web-based interventions to specific target groups [44, 45]. Within “GET.ON Stress”, the personas aim to increase user engagement in the training, providing knowledge about how to complete the exercises within the training and helping users to transfer what they learn from the exercises into their daily lives; for instance, by giving examples of how employees working under similar circumstances apply a given problem-solving strategy in their daily life. Examples of the adapted personas for the MSE context can be found in Additional file 1. In addition to training content, participants can receive written feedback from an e-coach on their exercises after each training session. E-coaches are psychotherapists or master’s degree-level psychologists. Based on our experience from previous studies, we anticipate that the e-coaches will spend roughly 30 min per feedback. To improve the adherence of the participants with the training, the e-coaches will send reminders to participants any time they fail to complete a training session within 7 days. All communication between the participant and the e-coach will take place anonymously within the secured web-based platform “System P”. The duration of the intervention is completely self-paced (between 6 weeks and 6 months).

**Table 2:** Contents of the web-based stress management training (SMT)

Session^**a**^	Intervention content
1	Psychoeducation on stress and coping competencies Enhancement of pleasant activities
2	Problem-solving I – identifying and differentiating solvable and unsolvable problems; developing an initial problem-solving plan Information and exercises on selected topics, which users can self-select^b^
3	Problem-solving II – self-evaluating the problem-solving plan; adapting or developing a new problem-solving plan Information and exercises on selected topics, which users can self-select^b^
4	Emotion regulation I – progressive muscle relaxation Information and exercises on self-selected topics^b^
5	Emotion regulation II – acceptance and tolerance of (negative) emotions Information and exercises on self-selected topics^b^
6	Emotion regulation III – effective self-support in times of stress Information and exercises on self-selected topics^b^
7	Developing a stress-coping plan for the future

Note: ^a^ each session lasts approximately 45 to 90 min; ^b^ optional exercises will cover the topics of time management, rumination and worrying, psychological detachment from work, sleep hygiene, the rhythm and regularity of sleeping habits, nutrition and exercise, organization of breaks during work, and social support

### Implementation strategy for the web-based platform “System P”

The web-based tool for PRA and the web-based SMT form the basis for the integrated web-based platform “System P”. Next to these two intervention components, “System P” also contains further components that are part of the implementation strategy. To overcome lack of knowledge, one of the barriers of stress prevention implementation in MSE, the platform provides educational information about stress and prevention in a web-based “stress lexicon”. Typical obstacles in the implementation of stress prevention in MSE and recommendations for their solution are additionally described in the frequently asked questions section (FAQ). The platform includes a number of short videos and audio instructions throughout. To further facilitate the implementation process, employers have access to a moderated forum where they can exchange information on their implementation process with other employers of MSE. All components of the platform can be accessed via the central “cockpit” page after login, which also includes a tutorial video that introduces all components. The design of the platform uses neutral colors (blue, grey, orange) and illustrations rather than photos to account for the heterogeneity of MSE across occupational sectors.

The initial steps of the implementation strategy are applied on the public project website, which has been developed accordingly to increase adoption: On this landing page, which is accessible without registration, the platform includes a short introduction video, application examples, three self-tests (checklist for prevention activities of company, questionnaire on personal stress level, and quiz to test knowledge about stress) and an overview of the most important benefits of “System P”. The landing page also links to a one-hour introductory webinar, in which prevention experts from the project group will explain all components of “System P” as well as the required efforts and expected benefits. Employers of MSE can attend the live session or access the recording afterwards. To gain trust in the web-based platform, it will be advertised via inter-corporate stakeholders for occupational prevention, who are legally responsible for providing information on occupational health-prevention in Germany (more information on the recruitment procedure can be found in the following sections). Representatives of these stakeholders have also been involved in the development of the web-based platform by providing feedback on prototypes of “System P” to assess whether it matches the needs of MSE.

The main premise of” System P″ is to enable MSE to access, implement and evaluate the interventions independently without additional costs while at the same time receiving professional support on demand. The implementation strategy cannot be evaluated independently of the web-based platform.

## Part II -Effect and process evaluation of the web-based platform “System P”

### Study design

**Fig. 1:**
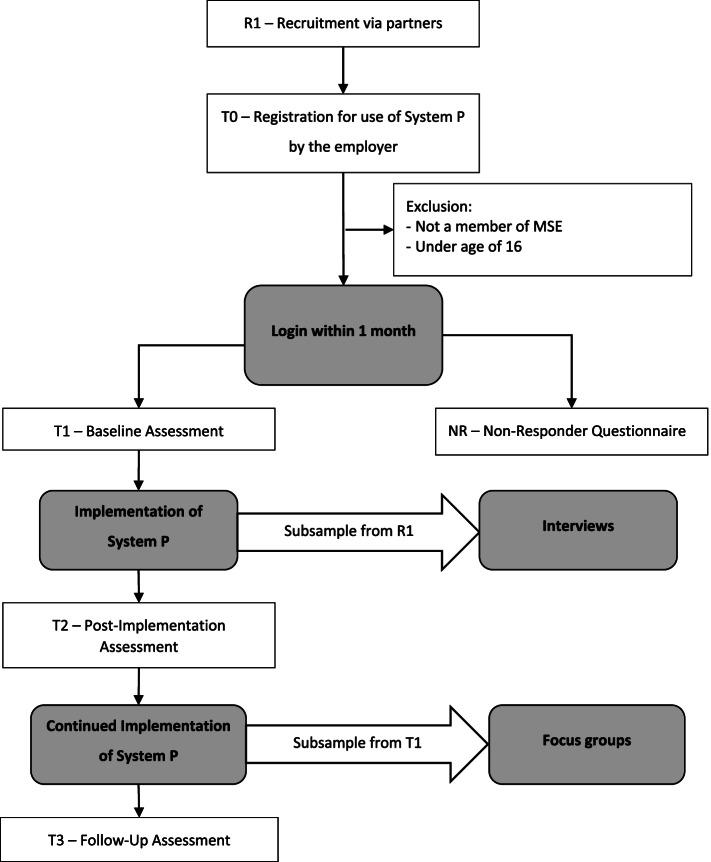
Study Flow

The presented study has a type 2 hybrid design for implementation research, evaluating the effectiveness and the implementation process of the intervention at the same time [46]. We will use a one-sample repeated measures design to evaluate the effectiveness of stress prevention via “System P”. Changes in mental health and work-related stress will be measured with the help of self-rated online questionnaires administered directly via the platform (see Table 3). Measurements will take place at baseline (T1), 6 months afterwards (T2) and 12 months after baseline (T3) (see Fig. 1). The primary outcome with regard to effectiveness will be the change in self-rated depressive symptoms between T1 and T2. Additional measurements for the process evaluation will take place between the assessment points [47], with semi-structured personal interviews between T1 and T2, and focus group interviews between T2 and T3. This study protocol describes the design of the pragmatic trial based on Standard Protocol Items: Recommendations for Interventional Trials (SPIRIT) guidelines [48] (see Additional file 2).

**Table 3:** Quantitative measures and assessment points

Questionnaire	T1	T2	T3	NR
Socio-demographic variables (age, gender, family status, first language, education, occupational position, working hours, previous contact with mental illness, income, affinity for technology), 11 items	✓	–	–	✓
Information about the organization (staff headcount, history of health program use in the organization, location, occupational sector), 4 items	✓			✓
Patient Health Questionnaire (PHQ-8), 8 items	✓	✓	✓	
Perceived Stress Scale (PSS-4), 4 items	✓	✓	✓	
Irritation (subscale cognitive irritation), 3 items	✓	✓	✓	
Psychosocial Safety Climate (PSC-4), 4 items	✓	✓	✓	
Work demands and support (COPSOQ), 7 items	✓	✓	✓	
Sickness absence and productivity (Tic-P), 6 items	✓	✓	✓	
User experience, 11 items		✓		
Readiness for change, 3 items	✓	✓	✓	✓
Harms, 1 item		✓	✓	

Note: T1 = Baseline Assessment; T2 = Post-Implementation Assessment, 6 months after T1; T2 = Follow-Up Assessment, 12 months after T1; NR = Non-Responder Questionnaire, 1 month after T0

### Participants

The studied population consists of employers and employees of MSE in Germany that are registered with regional and national stakeholders for occupational prevention. According to the European Commission, micro-sized enterprises are defined as enterprises with 1–9 employees and small-sized enterprises are defined as enterprises with 10–49 employees [49]. In total, more than 90% of all German enterprises are MSE with an average of five employees per organization [50]. Most of them are registered with at least one inter-corporate stakeholder for occupational prevention (e.g. occupational insurance association, company health insurance fund, etc). Participants are excluded from the study if they are under 16 years old or are not employers or employees in a MSE as defined above. As the present study concerns the implementation of stress prevention interventions in occupational practice, there are no further exclusion criteria.

### Recruitment procedure

Based on the results from preparatory studies, we developed a strategy to address and activate managers of MSE. We developed a joint communication strategy with cooperating recruitment partners (i.e., institutions and networks who support the occupational health and safety activities of MSE).

The first principle of this strategy is the digital distribution of information via email. The second principle of the strategy is cooperation with recruitment partners. Addressing employers of MSE via email is suitable, as it reaches a large sample with limited use of resources. Previous studies of the project show that employers typically obtain information on occupational health and safety topics from well-known networks and institutions, such as accident and health insurance companies. Hence, the access and communication strategy requires a close cooperation with these stakeholders (i.e. recruitment partners). We ask recruitment partners to contact MSE via email and inform them about the offer. Interested employers can follow a link to the project website, where they will learn about the advantages of stress prevention and “System P”. Each recruitment partner receives an individualized link, which allows a partner-specific analysis of the response behavior. Through the networks of our recruitment partners, we will be able to contact at least 1000 employers of MSE via email. As the recruitment partners will distribute the emails, the study team will not have access to any personal information prior to registration (e.g. names and email addresses).

Employers who fulfil the inclusion criteria will get access to the platform. They will also receive information about how to invite their employees to participate in the use of the platform. Both employers and employees can access the platform via personal accounts after a formal registration and acceptance of the terms and conditions for study participation and data protection. Employers of MSE that fulfil the inclusion criteria at registration (T0) but do not use the platform further (no login within 1 month) will be contacted again via email to fill out a short non-responder questionnaire about their socio-demographics and their reasons for not participating. To maximize the response rate, we will send a reminder via email for each questionnaire.

### Measurements

#### Primary outcome

Depression: The primary outcome measure is depression measured by the short version of the Patient Health Questionnaire (PHQ-8) at individual level [51]. The PHQ-8 contains eight items on depressive symptoms asking how often these occurred within the past 2 weeks with answers ranging from 0 “not at all” to 3 “almost every day”. The total score ranges between 0 and 24. The PHQ-scale has a good internal consistency and is sensitive to changes in mental health in clinical and non-clinical samples [52, 53].

#### Secondary outcomes

Perceived Stress: To measure the extent to which participants experience their lives as stressful we will use a German adaptation of the short version of the Perceived Stress Scale (PSS-4 [54];). It consists of four items asking respondents to rate how often they experienced stressful situations in the previous month on a five-point Likert scale ranging from 0 “never” to 4 “very often”. The scale has acceptable psychometric properties [54].

Psychosocial Safety Climate: Psychosocial Safety Climate will be measured by a German adaptation of the short version of the Psychosocial Safety Climate Questionnaire (PSC-4 [55–57];). It encompasses four items on management commitment and support, management priority, organizational commitment, and participation. Items are rated on a five-point Likert scale from 1 “strongly disagree” to 5 “strongly agree”. The scale exhibits good internal reliability and relates to a range of work conditions, health and engagement outcomes [55].

Irritation: Perceived cognitive tenseness will be assessed using the cognitive irritation subscale of the Irritation Scale, a reliable and valid measure of work-related stress that has been used across different industries and cultures [58]. The cognitive irritation subscale consists of three items on the ability to relax after work which are evaluated on a Likert scale ranging from 1 “completely disagree” to 7 “completely agree”.

Working conditions: To assess the general working conditions, we will use seven items of the German version of the Copenhagen Psychosocial Questionnaire (COPSOQ [59, 60];). Five items about working demands (e.g. “do you have to work overtime?”) and control (e.g. “can you influence the amount of work assigned to you?”) and two items on social support in the workplace (e.g. “how often do you get help from your colleagues?”) will be rated by the participants on a five-point Likert scale from 1 “always” to 5 “never”. The COPSOQ shows good reliability and validity and can by administered across a wide range of professions and industries [60].

Sickness absence and productivity loss: A German version of the second part of the questionnaire on healthcare consumption and productivity losses for patients with a psychiatric disorder (TiC-P [61];) will be used to assess sickness absenteeism and sickness presenteeism. Absence from work will be measured by three items asking for short-term and long-term absence from work. Three items assess productivity losses due to reduced efficiency during paid work while sick. The scale is a feasible and reliable instrument to assess sickness absenteeism and sickness presenteeism [61].

Harms: To assess the possibility of negative side effects of the interventions, we include one item from the Implementation Outcome Scales for Digital Mental Health (iOSDMH) [62] on harms (“This program does not result in negative side effects.”) in the follow-up questionnaires at T2 and T3.

#### Process outcomes

Acceptability: One measurement of acceptability of stress prevention interventions is the readiness for change which will be assessed by three items adapted from Hoek et al. [63]. Items are “I believe in the value of stress prevention”, “Stress prevention is positive for the organization” and “Stress prevention is necessary” and the answers range from 1 “strongly disagree” to 5 “strongly agree”. After the implementation period, we will also measure satisfaction with the intervention by asking participants to rank the usefulness of each intervention component at T2 on a 5-point scale, with a target average score of 3 or higher.

Adoption/Reach: The proportion of MSE who adopted “System P” will be calculated by dividing the number of registered MSE at T0 by the number of invited MSE during recruitment (R1). Reach will be analyzed by comparing socio-demographic characteristics of MSE that implemented “System P” with the characteristics of non-responders (using aggregated data from the recruitment partners and data from non-responder questionnaire) to see if they are representative of the population.

Appropriateness: To gain further information on appropriateness (perceived fit for the setting), we will conduct semi-structured personal interviews with employers of MSE that are registered with inter-corporate stakeholders who are unable to contact their members via email newsletters at R1 as described above. Employers will be contacted via phone by the recruitment partners and referred to the project team. They will receive access to videos sequences introducing the web-based platform in the week prior to the interview and will then be asked about aspects that they liked and disliked as well as potential barriers for the use of the platform (the interview guide can be found in Additional file 3). The platform should be regarded as appropriate by a majority of the employers.

Fidelity: To measure fidelity, we analyze usage data collected directly via “System P”. We will track data on the times of login, number of steps completed in the web-based tool for PRA (minimum 2) and the number of completed sessions in the web-based training (minimum 5). Additionally, we will ask the participants about their use of the interventions in the online questionnaires at T2 and T3 (e.g. “How much time have you spent on average for the training?”).

Costs: The direct costs of the implementation in “System P” are none because participating organizations will not be charged for the use of the web-based platform during the study. Indirect costs will be calculated by multiplying the time spent on the platform by the average hourly salary. The hours spent logged in to the web-based platform will be monitored for employers, employee and e-coaches.

Feasibility & Sustainability: Feasibility and sustainability will be assessed by means of focus groups with a subsample of the participating MSE. All employers registered at T0 will receive an invitation to take part in the discussion about stress prevention and will be asked to describe their experience with “System P”. They will be asked to discuss barriers and facilitators for long-term implementation in their organization and suggest improvements to the web-based interventions. We will also monitor the proportion of MSE that still use the platform between T2 and T3, with a target of 20% or more.

Penetration: Penetration of the intervention within the organization will be analyzed comparing the number of registered employees who filled out the questionnaire at T1 with the number of employees reported by the employer at T1 with a target participation rate of 50% or more. We will also ask MSE during the focus groups whether new employees have received access to the platform after the implementation period to gain more information on the penetration of the interventions.

#### Potential moderators

We will explore potential moderating variables of the effectiveness of the stress prevention interventions and the implementation process: socio-demographic characteristics, experience with stress prevention and mental health problems, user experience and affinity for technology. To assess user experience, we will use the safety subscale of the System Usability Scale (Brooke 1996) and the questionnaire for modular evaluation based on the components model of user experience (meCUE) [64]. Affinity for technology is measured with one item of the Affinity for Technology Interaction Short Scale (ATI-S [65];).

### Analysis

#### Sample size

Power analysis has been performed with G*Power [66] for a one sample case on the primary outcome measure depressive symptoms (PHQ score). Based on the average changes in depression reported in previous research [19, 22], we estimate a medium effect size (Cohen’s d = 0.4) [67]. With a power of 0.95 and a one-sided alpha α of 0.05, this resulted in a sample size of *n* = 70 at post-intervention measurement (T2). Due to interdependencies of the observations within MSE, we multiplied n with the design effect (DE) of cluster-randomized trials (DE = 1+ (NC-1)*ICC) [68], assuming an average cluster size (NC) of 5 (average number of employees in German MSE) and an intra-class correlation coefficient (ICC) of 0.1. This results in a required sample size of *n* = 98, and a minimum number of 20 MSE. If only 2-person MSE register (NC = 2), this would result in a required sample size of *n* = 77, equaling 39 MSE. We cannot predict the actual size of the MSE (with a range from 1 to 49 employees) and how many MSE will complete post-intervention assessment (T2) but previous studies show that only one quarter of all MSE that start interventions for stress prevention complete them [69]. The goal is therefore to recruit between 80 and 150/200 MSE for the baseline measurement. In the case of more registrations, only the first 200 MSE will be considered for the subsequent analyses.

#### Analysis of quantitative data

To investigate the effectiveness of “System P”, the change in depressive symptoms between baseline and post-intervention assessment (T2) will be analyzed. Additionally, we will compare the change scores between individuals who completed a minimum amount of steps in the interventions (2 out of 3 steps for the PRA and 5 out of 7 steps in the SMT) and those who completed one step or less of the interventions. The effect evaluation will be performed with multilevel analysis, taking into account interdependencies at organizational level. For the quantitative process outcomes, we will calculate the average scores (e.g. for readiness for change) and average rates (e.g. penetration) and compare them across MSE with different socio-demographic characteristics.

#### Analysis of qualitative data

Qualitative data will be analyzed by transcribing the recordings and applying content analysis as described by Elo et al. [70]. It enables researchers to retrieve replicable and valid interferences with a dynamic categorization system. The initial categories will be based on the literature and the research questions (deductive approach) and can be expanded during the coding (inductive approach). Additionally, the category system and coding will be reviewed by two independent researchers. The results of the content analysis will also be summarized according to the Consolidated Framework for Implementation Research (CFIR) [71], which defines five main categories of determinants for successful implementation: (1) intervention characteristics, (2) outer setting, (3) inner setting, (4) characteristics of the individuals involved and (5) process of the implementation.

## Discussion

The study outlined in this paper aims to conduct a comprehensive evaluation of the implementation of stress prevention measures in MSE via the integrated web-based platform “System P”. “System P” combines a structural intervention (web-based tool for PRA) and behavioral intervention (web-based SMT) within a number of interactive features and additional information to overcome known barriers to stress prevention implementation. The analysis will include an effect evaluation and an extensive process evaluation to determine the success of the interventions under real-world conditions. Quantitative measurements will be complemented by qualitative data to gain more in-depth knowledge about the adequacy of the interventions and long-term success of the implementation. To the best of our knowledge, this will be the first implementation study for web-based stress prevention targeted at MSE.

One strength of the study design introduced is its variety in outcome measures. The success of the intervention is going to be assessed subjectively with standardized questionnaires filled in by employers as well as their employees, objectively by means of response rates, and also qualitatively by using interviews. This variety of measurement perspectives might help to foster the understanding of successful stress interventions in MSE.

Due to a restricted number of previous studies on the effectiveness of web-based tools for PRA, we are not sure about the expected effect sizes for the combined interventions. The present study design resembles an effectiveness-implementation hybrid design [46] which is considered a valuable alternative to the traditional research-practice divide in occupational health. The presented study can be classified as type 2 hybrid implementation research, whereby the evaluation of effectiveness and evaluation of the implementation process are of equal importance. This type of research considers established interventions in a new setting or target group, which in our case will be MSE. We hypothesize that a successful implementation process increases the effectiveness of web-based interventions for stress prevention. We also hope to identify additional drivers and barriers in the implementation process to adjust future interventions to the specific needs of MSE.

### Study limitations

One limitation of the study is the lack of a formal control group. Due to the high heterogeneity and low implementation rate among MSE, matching the enterprises adequately poses a major challenge. We will therefore compare those MSE who completed the necessary steps for the intervention with those who did not get far in their implementation process.

Another restriction lies in the chosen method of recruitment. The number of MSE registered with the cooperating recruitment partners is limited and the response rate to the invitation emails is likely to be low. In case we are unable to reach the required sample size for the implementation study with the proposed communication strategy, we will deploy a back-up strategy that includes alternative ways to recruit MSE. The project team will prepare target-group-oriented material for advertisements in magazines, social media campaigns, and personal recommendations to support the recruitment with or without the partners. Despite increasing the chances of successful recruitment, this unstructured communication strategy will restrict the opportunity to analyze adoption and reach of the implementation.

Even though we aim to reduce stigmatized terms and to promote the acceptance of work-related stress within the “System P”, the response behavior of the participants might be affected due to social desirability [72] and fear of stigmatization [73]. Talking about stress and mental health problems in the workplace is still a major challenge. In particular, employees may be concerned about negative consequences [74] and therefore may not answer honestly the online questionnaires or refuse to participate in the workplace check. Further, social desirability and the fear of stigmatization could lead to unexplained dropouts.

## Conclusion

Implementation rates of interventions for occupational stress prevention are lower in small enterprises. “System P” is an integrated web-based platform targeted specifically at the needs of MSE by providing low-cost and easy-to-access structural and behavioral interventions. The proposed evaluation process will gain valuable insight about the implementation process and help to improve the effectiveness of preventive efforts in a greater variety of organizational settings.

## Supplementary Information

**Additional file 1.** Personas integrated in the adapted web-based stress management training “GET.ON Stress”.

**Additional file 2.** SPIRIT 2013 Checklist: Recommended items to address in a clinical trial protocol and related documents.

**Additional file 3.** Interview guides.

## Data Availability

Screenshots of “System P”, self-developed items in the questionnaire and full interview guides are available on reasonable request. Guidelines for data monitoring, original informed consent materials and dissemination plans are available in German upon request. For these purposes, please contact the corresponding author (miri.engels@hhu.de). For further information about the collaborative research project PragmatiKK, please contact the project coordination (pragmatikk@hhu.de).

## Abbreviations

GDA: Gemeinsame Deutsche Arbeitsschutzstrategie (Joint German Occupational Safety and Health Strategy)
MSE: Micro and Small-Sized Enterprises
OHS: Occupational Health and Safety
PRA: Psychosocial Risk Assessment
SMT: Stress Management Training
SPIRIT: Standard Protocol Items: Recommendations for Interventional Trials

## References

[1] Wedegaertner F, Arnhold-Kerri S, Sittaro N-A, Bleich S, Geyer S, Lee WE (2013). Depression- and anxiety-related sick leave and the risk of permanent disability and mortality in the working population in Germany: a cohort study. BMC Public Health.

[2] Koopmans PC, Bültmann U, Roelen CAM, Hoedeman R, van der Klink JJL, Groothoff JW (2011). Recurrence of sickness absence due to common mental disorders. Int Arch Occup Environ Health.

[3] Ahola K, Virtanen M, Honkonen T, Isometsä E, Aromaa A, Lönnqvist J (2011). Common mental disorders and subsequent work disability: a population-based Health 2000 Study. J Affect Disord.

[4] Theorell T, Hammarstrom A, Aronsson G (2015). A systematic review including meta-analysis of work environment and depressive symptoms. BMC Public Health.

[5] Madsen IEH, Nyberg ST, Magnusson Hanson LL (2017). Job strain as a risk factor for clinical depression: Systematic review and meta-analysis with additional individual participant data. Psychol Med.

[6] Beck D, Lenhardt U, Schmitt B, Sommer S (2015). Patterns and predictors of workplace health promotion: cross-sectional findings from a company survey in Germany. BMC Public Health.

[7] Health and Safety Executive (HSE) (2014). Risk assessment: A brief guide to controlling risks in the workplace.

[8] Janetzke H, Ertel M. Psychosocial Risk Management in a European Comparison. Bundesanstalt für Arbeitsschutz und Arbeitsmedizin (BAuA). 2017; Available from: URL: http://www.baua.de/dok/8565216. Accessed 19 Oct 2021.

[9] Klenke B, Ghadiri A, Ternès A, Peters T (2016). Psychische Gefährdungsbeurteilungen in deutschen Unternehmen – Anforderungen, aktueller Stand und Vorgehensweisen. Trends im Betrieblichen Gesundheitsmanagement: Ansätze aus Forschung und Praxis.

[10] Beck D, Richter G, Ertel M, Morschhäuser M (2012). Gefährdungsbeurteilung bei psychischen Belastungen in Deutschland: Verbreitung, hemmende und fördernde Bedingungen. Praev Gesundheitsf.

[11] van Stolk C, Staetsky L, Hassan E, Woo C (2012). Management of psychosocial risks at work: An analysis of the findings of the European Survey of Enterprises on New And Emerging Risks (ESENER) ; European Risk Observatory report.

[12] Harris JR, Hannon PA, Beresford SAA, Linnan LA, McLellan DL (2014). Health promotion in smaller workplaces in the United States. Annu Rev Public Health.

[13] Wulf IC, Süß S, Diebig M (2017). Akteure der Gefährdungsbeurteilung psychischer Belastung – Perspektiven und Konflikte im betrieblichen Arbeits- und Gesundheitsschutz. Z Arb Wiss..

[14] Pavlista V, Angerer P, Diebig M (2021). Barriers and drivers of psychosocial risk assessments in German micro and small-sized enterprises: a qualitative study with owners and managers. BMC Public Health.

[15] Cunningham TR, Sinclair R, Schulte P (2015). Better understanding the small business construct to advance research on delivering workplace health and safety. Small Enterp Res.

[16] Beck D, Lenhardt U (2019). Consideration of psychosocial factors in workplace risk assessments: findings from a company survey in Germany. Int Arch Occup Environ Health.

[17] da Silva SLC, Amaral FG (2019). Critical factors of success and barriers to the implementation of occupational health and safety management systems: A systematic review of literature. Saf Sci.

[18] Heber E, Ebert DD, Lehr D (2017). The Benefit of Web- and Computer-Based Interventions for Stress: A Systematic Review and Meta-Analysis. J Med Internet Res.

[19] Havermans BM, Boot CR, Brouwers EP (2018). Effectiveness of a digital platform-based implementation strategy to prevent work stress in a healthcare organization: A 12-month follow-up controlled trial. Scand J Work Environ Health.

[20] Diebig M, Dragano N, Körner U, Lunau T, Wulf IC, Angerer P (2020). Development and Validation of a Questionnaire to Measure Psychosocial Work Stressors in Modern Working Environments. J Occup Environ Med.

[21] Dragano N, Wulf IC, Diebig M. *digitale* Gefährdungsbeurteilung psychischer Belastung. In: Fehlzeiten-Report 2019. Berlin: Springer; 2019. p. 111–25.

[22] Phillips EA, Gordeev VS, Schreyögg J. Effectiveness of occupational e-mental health interventions: a systematic review and meta-analysis of randomized controlled trials. Scand J Work Environ Health. 2019; 10.5271/sjweh.3839. [PMID: 31184758] 10.5271/sjweh.3839 31184758

[23] Ebert DD, Kählke F, Buntrock C (2018). A health economic outcome evaluation of an internet-based mobile-supported stress management intervention for employees. Scand J Work Environ Health.

[24] Carolan S, Harris PR, Cavanagh K (2017). Improving Employee Well-Being and Effectiveness: Systematic Review and Meta-Analysis of Web-Based Psychological Interventions Delivered in the Workplace. J Med Internet Res.

[25] Peters DH, Adam T, Alonge O, Agyepong IA, Tran N (2013). Implementation research: What it is and how to do it. BMJ.

[26] Nielsen K, Randall R (2013). Opening the black box: Presenting a model for evaluating organizational-level interventions. Eur J Work Organ Psychol.

[27] Proctor E, Silmere H, Raghavan R (2011). Outcomes for implementation research: conceptual distinctions, measurement challenges, and research agenda. Adm Policy Ment Health.

[28] Davis FD (1993). User Acceptance of Information Technology: System Characteristics, User Perceptions and Behavioral Impacts. Int J Man-Machine Stud.

[29] Rabin BA, Brownson RC, Haire-Joshu D, Kreuter MW, Weaver NL (2008). A glossary for dissemination and implementation research in health. J Public Health Manag Pract.

[30] Karsh B-T (2004). Beyond usability: designing effective technology implementation systems to promote patient safety. Qual Saf Health Care.

[31] Stiles PG, Boothroyd RA, Snyder K, Zong X (2002). Service penetration by persons with severe mental illness: how should it be measured?. J Behav Health Serv Res.

[32] Pluye P, Potvin L, Denis J-L (2004). Making public health programs last: conceptualizing sustainability. Eval Program Plan.

[33] McCoy K, Stinson K, Scott K, Tenney L, Newman LS (2014). Health promotion in small business: a systematic review of factors influencing adoption and effectiveness of worksite wellness programs. J Occup Environ Med.

[34] Gemeinsame Deutsche Arbeitsschutzstrategie. Empfehlungen zur Umsetzung der Gefährdungsbeurteilung psychischer Belastung. Berlin: Arbeitschutz in der Praxis; 2017. Available online at: https://www.gda-psyche.de/SharedDocs/Downloads/DE/empfehlungen-zur-umsetzung-der-gefaehrdungsbeurteilung-psychischer-belastung.pdf?__blob=publicationFile&v=1. Last retrieved on 19 Oct 2021.

[35] Diebig M, Angerer P (2021). Description and application of a method to quantify criterion-related cut-off values for questionnaire-based psychosocial risk assessment. Int Arch Occup Environ Health.

[36] Heber E, Lehr D, Ebert DD, Berking M, Riper H (2016). Web-Based and Mobile Stress Management Intervention for Employees: A Randomized Controlled Trial. J Med Internet Res.

[37] Ebert DD, Lehr D, Heber E, Riper H, Cuijpers P, Berking M (2016). Internet-and mobile-based stress management for employees with adherence-focused guidance: Efficacy and mechanism of change. Scand J Work Environ Health.

[38] Ebert DD, Heber E, Berking M (2016). Self-guided internet-based and mobile-based stress management for employees: results of a randomised controlled trial. Occup Environ Med.

[39] Nixon P, Boß L, Heber E, Ebert DD, Lehr D (2021). A three-armed randomised controlled trial investigating the comparative impact of guidance on the efficacy of a web-based stress management intervention and health impairing and promoting mechanisms of prevention. BMC Public Health.

[40] D'Zurilla TJ, Nezu AM, Dobson KS (2010). Problem-solving therapies. Handbook of cognitive-behavioral therapies.

[41] Berking M, Whitley B (2014). Affect Regulation Training: A Practitioners’ Manual.

[42] Heber E, Ebert DD, Lehr D, Nobis S, Berking M, Riper H (2013). Efficacy and cost-effectiveness of a web-based and mobile stress-management intervention for employees: design of a randomized controlled trial. BMC Public Health.

[43] Cooper A, Reimann R, Cronin D (2007). About Face 3: The Essentials of Interaction Design.

[44] Holden RJ, Kulanthaivel A, Purkayastha S, Goggins KM, Kripalani S (2017). Know thy eHealth user: Development of biopsychosocial personas from a study of older adults with heart failure. Int J Med Inform.

[45] Serio CD, Hessing J, Reed B, Hess C, Reis J (2015). The effect of online chronic disease personas on activation: within-subjects and between-groups analyses. JMIR Res Protoc.

[46] Landes SJ, Mcbain SA, Curran M (2019). Reprint of : An introduction to effectiveness-implementation hybrid designs. Psychiatry Res.

[47] Hamilton AB, Finley EP. Qualitative methods in implementation research: An introduction. Psychiatry Res. 2019:280. 10.1016/j.psychres.2019.112516. 10.1016/j.psychres.2019.112516 PMC7023962 31437661

[48] Chan A-W, Tetzlaff JM, Gøtzsche PC (2013). SPIRIT 2013 explanation and elaboration: guidance for protocols of clinical trials. BMJ.

[49] European Commission (2003). Commission Recommendation of 6 May 2003 concerning the definition of micro, small and medium-sized enterprises.

[50] Statistisches Bundesamt (Destatis). Kleine und mittlere Unternehmen; 2021 [cited 2021 June 16] Available from: URL: https://www.destatis.de/DE/Themen/Branchen-Unternehmen/Unternehmen/Kleine-Unternehmen-Mittlere-Unternehmen/_inhalt.html;jsessionid=2DF8779B9BB68114C815B8892DECA138.live741#sprg475846. Accessed 19 Oct 2021.

[51] Kroenke K, Strine TW, Spitzer RL, Williams JBW, Berry JT, Mokdad AH. The PHQ-8 as a measure of current depression in the general population. J Affect Disord 2009; 114(1–3): 163–73 [10.1016/j.jad.2008.06.026][PMID: 18752852]. 10.1016/j.jad.2008.06.026 18752852

[52] Löwe B, Unützer J, Callahan CM, Perkins AJ, Kroenke K (2004). Monitoring Depression Treatment Outcomes with the Patient Health Questionnaire-9. Medical care.

[53] Phillips R, Schneider J, Molosankwe I (2014). Randomized controlled trial of computerized cognitive behavioural therapy for depressive symptoms: effectiveness and costs of a workplace intervention. Psychol Med.

[54] Warttig SL, Forshaw MJ, South J, White AK (2013). New, normative, English-sample data for the Short Form Perceived Stress Scale (PSS-4). J Health Psychol.

[55] Dollard MF, Dollard MF, Dormann C, Awang Idris M (2019). The PSC-4; A Short PSC Tool. Psychosocial Safety Climate: A New Work Stress Theory.

[56] Otto W, Neuert C, Meitinger K, Beitz C, Schmidt R, Stiegler A. Psychosocial Safety Climate - Weiterentwicklung und Validierung eines Instrumentes für die Erfassung der Handlungsbereitschaft zum Schutz der psychischen Gesundheit der Beschäftigten auf organisationaler Ebene. Kognitiver Pretest. GESIS Projektbericht. Mannheim. Version: 1.0. GESIS - Pretestlabor. Text. 2016. Available online at: 10.17173/pretest38.

[57] Berthelsen H, Muhonen T, Bergström G, Westerlund H, Dollard MF (2020). Benchmarks for Evidence-Based Risk Assessment with the Swedish Version of the 4-Item Psychosocial Safety Climate Scale. Int J Environ Res Public Health.

[58] Mohr G, Rigotti T, Müller A (2005). Irritation - ein Instrument zur Erfassung psychischer Beanspruchung im Arbeitskontext. Skalen- und Itemparameter aus 15 Studien. Zeitschrift für Arbeits- und Organisationspsychologie A&O.

[59] Kristensen TS, Hannerz H, Høgh A, Borg V (2005). The Copenhagen Psychosocial Questionnaire--a tool for the assessment and improvement of the psychosocial work environment. Scand J Work Environ Health.

[60] Nübling M, Stößel U, Hasselhorn H-M, Michaelis M, Hofmann F (2006). Measuring psychological stress and strain at work - Evaluation of the COPSOQ Questionnaire in Germany. Psychosoc Med.

[61] Bouwmans C, Jong K de, Timman R*, et al.* Feasibility, reliability and validity of a questionnaire on healthcare consumption and productivity loss in patients with a psychiatric disorder (TiC-P). BMC Health Serv Res 2013; 13(1): 217. [10.1186/1472-6963-13-217]. [PMID: 23768141]. 10.1186/1472-6963-13-217 PMC3694473 23768141

[62] Sasaki N, Obikane E, Vedanthan R (2021). Implementation Outcome Scales for Digital Mental Health (iOSDMH): Scale Development and Cross-sectional Study. JMIR Form Res.

[63] Hoek RJA, Havermans BM, Houtman ILD (2018). Stress Prevention@Work: a study protocol for the evaluation of a multifaceted integral stress prevention strategy to prevent employee stress in a healthcare organization: a cluster controlled trial. BMC Public Health.

[64] Minge M, Thüring M, Wagner I, Kuhr CV, Soares M, Falcão C, Ahram TZ (2016). The meCUE Questionnaire: A Modular Tool for Measuring User Experience. The meCUE Questionnaire: A Modular Tool for Measuring User Experience.

[65] Wessel D, Attig C, Franke T, Alt F, Bulling A, Döring T (2019). ATI-S - An Ultra-Short Scale for Assessing Affinity for Technology Interaction in User Studies. ATI-S - An Ultra-Short Scale for Assessing Affinity for Technology Interaction in User Studies.

[66] Faul F, Erdfelder E, Lang A-G, Buchner A (2007). G*Power 3: a flexible statistical power analysis program for the social, behavioral, and biomedical sciences. Behav Res Methods.

[67] Cook JA, Julious SA, Sones W (2018). DELTA2 guidance on choosing the target difference and undertaking and reporting the sample size calculation for a randomised controlled trial. Trials.

[68] Lorenz E, Köpke S, Pfaff H, Blettner M (2018). Cluster-Randomized Studies. Dtsch Arztebl Int.

[69] Beck D (2019). Psychische Belastung als Gegenstand des Arbeitsschutzes. Arbeit.

[70] Elo S, Kyngäs H (2008). The qualitative content analysis process. J Adv Nurs.

[71] Damschroder LJ, Aron DC, Keith RE, Kirsh SR, Alexander JA, Lowery JC (2009). Fostering implementation of health services research findings into practice: a consolidated framework for advancing implementation science. Implement Sci.

[72] Podsakoff PM, Organ DW (1986). Self-Reports in Organizational Research: Problems and Prospects. J Manag.

[73] Wheat K, Brohan E, Henderson C, Thornicroft G (2010). Mental illness and the workplace: conceal or reveal?. J R Soc Med.

[74] Jones AM (2011). Disclosure of Mental Illness in the Workplace: A Literature Review. American Journal of Psychiatric Rehabilitation.

