# Supplementary material for: Facilitating stress prevention in micro and small-sized enterprises: protocol for a mixed method study to evaluate the effectiveness and implementation process of targeted web-based interventions
Source: BMC Public Health. 2022 Mar 26;22:591. doi: 10.1186/s12889-022-12921-7 (PMC8959270; doi:10.1186/s12889-022-12921-7)
Supplement: Supplementary file 1 — Additional file 1. Personas integrated in the adapted web-based stress management training “GET.ON Stress”. [file 12889_2022_12921_MOESM1_ESM.docx]

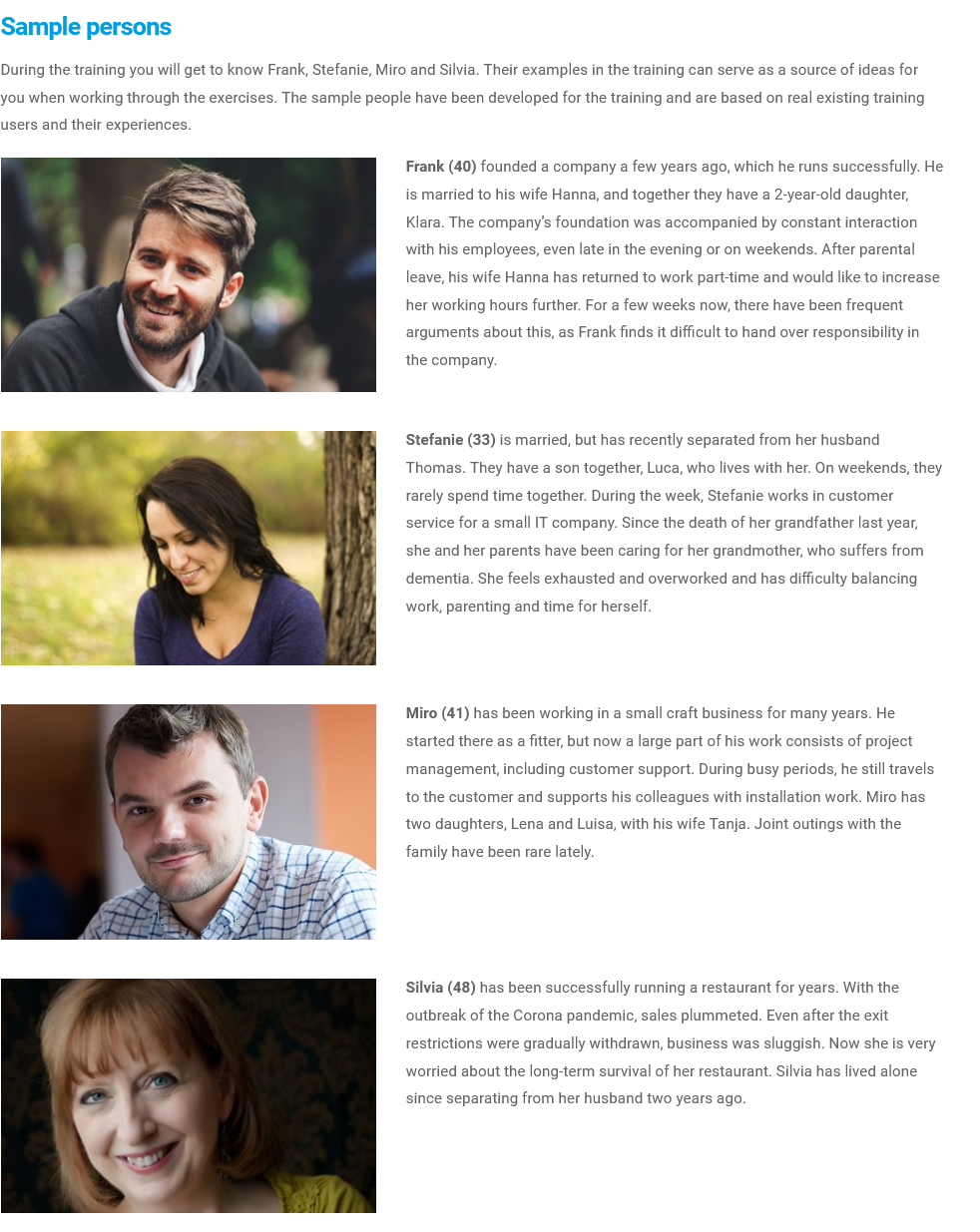


Additional file 1. Personas integrated in the adapted web-based stress management training “GET.ON Stress”
