# Supplementary material for: Facilitating stress prevention in micro and small-sized enterprises: protocol for a mixed method study to evaluate the effectiveness and implementation process of targeted web-based interventions
Source: BMC Public Health. 2022 Mar 26;22:591. doi: 10.1186/s12889-022-12921-7 (PMC8959270; doi:10.1186/s12889-022-12921-7)
Supplement: Supplementary file 3 — Additional file 3. Interview guides. [file 12889_2022_12921_MOESM3_ESM.docx]

Additional file 3. Interview guides

**Personal Interview Guide**

| **Main Question** | **Sub-Questions** | **Outcome** | **Determinants (CFIR)** |
| --- | --- | --- | --- |
| 1. Please describe your company. | 1. Type/Sector/Size |  | Inner Setting |
|  | 1. Role within company |  | Characteristics of Individuals |
|  | 1. Change and safety climate of company | Acceptability | Inner Setting |
| 1. Prior experience with stress prevention? | 1. Prior experience with PRA? |  | Inner Setting  Characteristics of Individuals |
|  | 1. Prior experience with SMT? |  | Inner Setting  Characteristics of Individuals |
| 1. What is your impression of System P? | 1. What do you like about it? | Acceptability | Intervention Characteristics |
|  | 1. What do you dislike about it? | Acceptability | Intervention Characteristics |
|  | 1. Would you recommend it to other owners of MSE? | Acceptability | Intervention Characteristics |
| 1. How well do you think System P can be implemented in your company? | 1. What are potential difficulties? | Appropriateness | Intervention Characteristics,  Inner Setting |
|  | 1. How do you rate the adaptability of the system to the needs of your company? | Appropriateness | Intervention Characteristics,  Inner Setting |
| 1. What benefits do you expect from System P? | 1. For your company? | Acceptability | Intervention Characteristics |
|  | 1. For your employees? | Acceptability | Intervention Characteristics |
|  | 1. For you personally? | Acceptability | Intervention Characteristics |
| 1. How do you assess the workplace check (PRA)? | 1. How helpful do you consider the workplace check? | Appropriateness | Intervention Characteristics |
|  | 1. How do you rate the workplace check in comparison with other PRA tools? | Appropriateness | Intervention Characteristics |
| 1. How do you assess the training (SMT)? | 1. How helpful do you consider the training? | Appropriateness | Intervention Characteristics |
|  | 1. How do you rate the “Get.ON Stress” training in comparison with other trainings? | Appropriateness | Intervention Characteristics |
| 1. How do you assess the forum? | 1. How do you rate the possibility of exchanging information with other employers? | Appropriateness | Intervention Characteristics,  Outer setting |
| 1. How do you assess the stress lexicon and FAQ? | 1. How useful do you rate the additional information in the web-based platform? | Appropriateness | Intervention Characteristics,  Outer setting |

**Focus Group Interview Guide**

| **Main Question** | **Sub-Question** | **Outcome** | **Determinants (CFIR)** |
| --- | --- | --- | --- |
| 1. Please describe your company. | 1. Type/Sector/Size |  | Inner Setting |
|  | 1. Role within company |  | Characteristics of Individuals |
|  | 1. Change and safety climate of company | Acceptability | Inner Setting |
| 1. Prior experience with stress prevention? | 1. Prior experience with PRA? |  | Inner Setting  Characteristics of Individuals |
|  | 1. Prior experience with SMT? |  | Inner Setting  Characteristics of Individuals |
| 1. How would you rate your experience with System P? | 1. What did you like about it? | Acceptability | Intervention Characteristics |
|  | 1. What did you dislike about it? What would you want to change? | Acceptability | Intervention Characteristics |
|  | 1. Would you recommend it to other owners of MSE? | Acceptability | Intervention Characteristics |
| 1. How well were you able to implement System P in your company? | 1. How did your employees react to the introduction of System P? | Feasibility  Penetration | Process of Implementation,  Inner Setting |
|  | 1. What were difficulties in the implementation process? | Feasibility | Process of Implementation,  Inner Setting |
|  | 1. What was helpful during the implementation process? | Feasibility | Process of Implementation,  Inner Setting, Outer Setting |
|  | 1. What are your suggestions for improvement of System P? | Feasibility  Appropriateness | Intervention Characteristics |
| 1. What benefits did you experience from the use of System P? | 1. For your company? | Acceptability | Intervention Characteristics |
|  | 1. For your employees? | Acceptability | Intervention Characteristics |
|  | 1. For you personally? | Acceptability | Intervention Characteristics |
| 1. How much time did you spend on the implementation of System P? | 1. How much time did you spend on the interventions online? | Dose/ Fidelity | Process of Implementation |
|  | 1. How much time did you spend on the interventions offline? | Dose/ Fidelity | Process of Implementation |
|  | 1. How did your use of System P change over time? | Sustainability | Process of Implementation |
| 1. What are your plans for using System P in the future? | 1. Would you like to keep access to System P? | Sustainability | Intervention Characteristics |
|  | 1. How are new employees informed about System P? | Penetration | Intervention Characteristics |
